# Homœopathy Repudiated in Law

**Published:** 1850-09

**Authors:** 


*Homœopathy repudiated in Law.*—A case recently occurred in the city of New York, in which the legal merits of homœopathy came up for judicial decision. An Italian opera-singer sued the manager of the operahouse for a balance of $100, for his services. To this a defence was made that the plaintiff had incurred a forfeiture of that amount, by failing to perform on a certain evening, and not giving notice of illness under the certificate of the Physician to the establishment, as the regulations required. The plaintiff proved by his own attending physician, who was a member of the regular profession, that on the night referred to, he was really ill and unable to perform. It appeared in evidence that the Physician to the opera-house, whose certificate of illness was required by the regulations of the establishment, was not a regular practitioner. Under these circumstances, Judge Lynch decided in favor of the plaintiff, on the ground that it was not proved that Dr. Quinn, the opera-house doctor, "was a doctor, or that he had taken a degree as Doctor of Medicine, or that he was authorized by the Medical Society, or had a regular license to practise, which was necessary in order to constitute him a doctor." The Judge adds: "So far as there is evidence on the subject, it went to show that Dr. Quinn practises upon principles of homœopathy, and that such practitioners are not recognized by the faculty of medicine, nor by a majority of the public, as regular practitioners." Such being the case, the Judge thought the evidence of illness afforded by the testimony of the attending regular physician sufficient to exculpate from the forfeiture, without imposing on the plaintiff the necessity of getting the certificate of the homœopathic practitioner, although the rules of the house required the latter.

